# Brazil nut journey under future climate change in Amazon

**DOI:** 10.1371/journal.pone.0312308

**Published:** 2024-11-13

**Authors:** Luciano J. S. Anjos, Gabriela S. R. Gonçalves, Vítor A. B. Dutra, Amanda G. Rosa, Lucyana B. Santos, Márcia N. R. Barros, Everaldo B. de Souza, Peter M. de Toledo

**Affiliations:** 1 Faculdade de Meteorologia, Instituto de Geociências, Universidade Federal do Pará—UFPA, Belém, Pará, Brazil; 2 Programa de Pós-Graduação em Ciências Ambientais, Instituto de Geociências, Universidade Federal do Pará—UFPA, Belém, Pará, Brazil; 3 Laboratório de Biogeografia da Conservação e Macroecologia, Instituto de Ciências Biológicas, Universidade Federal do Pará—UFPA, Belém, Pará, Brazil; 4 Programa de Pós-Graduação em Ciências Ambientais, Universidade do Estado do Pará—UEPA, Belém, Pará, Brazil; 5 Divisão de Impactos, Adaptação e Vulnerabilidade, Instituto Nacional de Pesquisas Espaciais—INPE, São José dos Campos, São Paulo, Brazil; Universidade Federal de Minas Gerais, BRAZIL

## Abstract

Climate change is among the principal threats to global terrestrial biodiversity, especially to megadiverse ecosystems such as the Amazon rainforest. In this study, we investigate how it could affect an iconic forest species—*Bertholletia excelsa—*(the Brazil nut) which has values in multiple dimensions in an Amazonian context. We used an ensemble from various distribution modeling methods designed for four different climate scenarios from CMIP6 by the end of the century. Then, we simulate how spatial dynamics under climate change, including explicitly dispersal events, can affect the persistence, colonization, and potential extinction of *Bertholletia excelsa* in the future. Our results show that by the end of the century there would be a generalized loss of suitability on the Amazon biome, regardless of the climate scenario evaluated, which could promote a significant loss (up to 94%) of the area available for the species via extinction. Our results also show that, in the future, the species would colonize higher altitudes in search of favorable conditions for its survival. Finally, we detected that areas that had previously become unsuitable because of climate change would have favorable conditions by the end of the century. Such an outcome could be useful in fostering an active restoration agenda that can mitigate the negative effects of climate change on species in this study.

## Introduction

Human-induced climatic change has been emphasized as a significant threat to biodiversity [1]. Climate change can cause effects along the various levels of biodiversity organization, such as on individual organisms, populations, communities, ecosystems, and biomes [2]. Such changes can affect the survival rate of species, alter their phenological dynamics and life history traits, as well as the network of interactions of communities, impacting on interspecific relationships and their structure [3].

In response to the changes, species move into geographic space, seeking adequate climatic conditions for their survival, growth, and reproduction [2, 4], changing their distribution patterns over generations [5, 6]. In this way, it can lead to population declines, or even species extinction [7, 8]. As consequence of these cascading events, changes in the climate pattern can promote the loss of biodiversity and ecosystem functions, such as seed dispersal, the germinative success of these seeds, and therefore the process of forest regeneration [9], affecting the integrity of biomes on a wide scale [10].

In the Amazon, the consequences of climate change can range from extreme weather events such as droughts and floods, changes in rainfall regime, increased fire risk associated with impacts on climate, health, and biodiversity [11, 12]. Furthermore, extreme droughts and floods cause additional stress on Amazonian forests, increasing the risk of tree mortality [13]. Thus, if changes reach a critical threshold because of increases in temperature, generating longer drought periods in the near future, major changes would be triggered in Amazonia, such as large-scale "savannization" events [11, 12, 14].

According to [15], continuous deforestation in the Amazon severely affected about 36 to 57% of all common and rare tree species. Inasmuch, some species are the merger of losing over 30% of their original population. This is the case of the Brazilian Nut tree (*Bertholletia excelsa*), an iconic species of the Amazon region that is among the vulnerable species, under the criteria of the International Union for Conservation of Nature (IUCN) Red List [16].

The Brazil nut tree is a long-lived species that is demanding in terms of favorable light for its survival and ascent in the canopy. In the seedling stage it is demanding for light, especially in open areas such as clearings and grazing, favoring its vertical growth, besides having a high capacity for regrowth and performance recovery after traumatic episodes of physiological nature, being considered indicators of past disturbances. Therefore, its life cycle is associated with natural events and disturbances such as extreme floods, droughts, tree fall gaps and fires [17–20].

*B*. *excelsa* is one of the most important non-timber products producing trees in the Amazon bioeconomy. Its fruits are predominantly harvested from natural forests. The species is an emergent tree, reaching heights up to 50 m, and is a long-lived and pioneer. It usually grows in areas above sea level (> 400 m) with an annual mean temperature of 23.5 to 27.6°C and annual rainfall ranging from 1,445 to 3,399 mm [21].

In this study, to analyze suitability conditions for the Brazil nut distribution by the end of the current century, we used species distribution modeling under future climatic conditions not analogous to the current ones. We also quantify over continuous geographical space and time in the Amazon landscape, under different climatic scenarios, the extinction patterns, persistence places, and colonization of potential distribution areas. Since the species at hand has a strong dependence on human consumption and traditional cultivation activities, this historical distribution pattern must be an issue to be addressed in the conservation strategies.

## Material and methods

### Area of study

The area of study comprises the Amazon biome in its biogeographic domains defined by the Amazon Network of Georeferenced Socio-Environmental Information (RAISG) polygon (http://raisg.socioambiental.org/ - accessed on the November 2023). The study area covers approximately 7 million km^2^. It is the world’s largest watershed, encompassing tropical rainforest and other types of vegetation [22]. The Amazon biome has been altered by deforestation and forest degradation, affecting 38% of the area [23].

### Current and future climate variables datasets

We used the bioclimatic variables of WorldClim 2.0 [24] (https://worldclim.org - accessed in 09/19/2023) as predictors with a spatial resolution of 0.0833° (~10 km). We selected the following variables: bio1—Average Annual Temperature, bio12—Annual cumulative precipitation; bio7—Annual temperature amplitude; and bio15—Seasonality of Precipitation. These predictors are often used for distribution models because of their relationship to species physiology and water/energy sources.

Regarding the simulated climate for the future, the bioclimatic variables mentioned above were downloaded from WorldClim 2.0 from the most recent simulation generated by CMIP6 (Coupled Model Intercomparison Project Phase 6). We used four distinct time periods until the end of the century: 2021–2040; 2041–2060; 2061–2080 and 2081–2100. To minimize the effect of uncertainty associated with the different Global Circulation Models (GCMs) [25], we used an average ensemble of five GCMs (BCC-CSM2-MR, CNRM-CM6-1, CNRM-ESM2-1, MIROC6, MIROC-ES2L). Such GCMs were selected considering the values of the Equilibrium Climate Sensibility (ECS) and Transition Climate Response (TCR) parameters [26].

Anthropogenic Greenhouse Gases (GHG) emissions are mainly driven by population size, economic activity, lifestyle, energy use, land use patterns, technology, and climate policy. In this sense, the Intergovernmental Panel for Climate Change (IPCC) conceptualized the SSPs—Shared Socioeconomic Pathways (SSPs) based on CMIP6 climate simulations [27]. We have selected four (04) simulated climate scenarios available on the WorldClim 2.0 (https://worldclim.org/data/) [28].

Among the scenarios used in this study, the most optimistic is SSP1-2.6, which points to a sustainable future where there are low challenges to mitigating and adapting to climate change [29]. We also use a scenario called Middle of the Road (SSP2-4.5), where the challenges of adapting to and mitigating climate change can be considered intermediate. Finally, we use two pessimistic scenarios: SSP3-7.0 (Regional Rivalry) and SSP5-8.5 (Fossil-fueled Development). The difference between them concerns the great challenges for adapting to climate change, where SSP3-7.0 points to high challenges in promoting adaptation. In other hand, SSP5-8.5, despite the economy and use of natural resources being based on the intensive use of fossil fuels, there would be more conditions for the generation and sharing of new adaptive technologies.

### Occurrence database

To prospect the species distribution of *Bertholletia excelsa* Bonpl., in the Amazon biome, we used geographical occurrences from the Global Biodiversity Information Facility (GBIF) (https://www.gbif.org/species/3083180; accessed in 16/05/2024), which is an international network and research infrastructure funded by the world’s governments and aimed at providing open access to data about much life on Earth. To download the occurrences, we used the ’dismo’ package [30]. After downloading the occurrences, we performed coordinate accuracy testing and duplicate exclusion with the ’biogeo’ package [31]. We compiled 255 unique occurrence records for Bertholletia excelsa, covering the entire area of study.

### Distribution model for *B*. *excelsa*

To build the species distribution model for *B*. *excelsa*, we used the package ’biomod2’ [32] implemented in the R software [33]. We built a consensus model based on an ensemble of seven different distribution modeling algorithms, which are: Artificial Neural Networks (ANN) [34]; Classification Tree Analysis (CTA) [35]; Function Discriminant Analysis (FDA) [34]; Generalized Additive Models (GAM) [36]; Generalized Boosted Regression Models (GBM) [37]; Generalized Linear Models (GLM) [38]; Random Forest (RF) [39].

For each modeling method, we ran 10 replicate models with 75–25% training-test proportion samples, respectively, re-sampled randomly by bootstrap. The 500 pseudo-absence samples were selected using the ’disk’ function, considering the limit of a radius of 1° (111.11 km) in the surroundings of each geographical occurrence for selection.

To evaluate the quality of the generated models, we used two metrics: True Skill Statistics (TSS) [40] and Receiving Operating Characteristic (ROC) [41] (S1 Table). For each model generated, a measure of the importance of climatic variables for the construction of the models was also computed (S2 Table). Finally, for ensemble composition, we selected only models with TSS values equal to or greater than 0.6. Our consensual distribution model, represented by the average model among the best rotated models, was then designed under present and future climatic conditions for the different scenarios and periods. The result comprises a measure of climate adequacy, ranging from inadequate (0) to the most appropriate (1000) climatically between the different periods and climate scenarios evaluated.

### Calculation of trend of gain or loss of climatic suitability

To assess the trend of changes in climate suitability for *Bertholletia excelsa* by the end of the century, we used the VoCC package [42] in R [33]. We use the ’tempTrend’ function that estimates coefficients of a linear regression and projects them over the geographic space, showing loss (negative values) or gain (positive values) of suitability for the species over the periods and climatic scenarios evaluated.

### Definition of the critical threshold of suitability for extinction

The distribution models have in their representation in a continuous form of suitability for the species. Here, ranging between zero (0) and 1000. One of the key points of the post-modeling steps is to define from which value along the gradient the species will be present (persistence or potential colonization) or absent (local extinction). As a critical threshold parameter for the extinction of the species in a pixel, we used the minimum training presence (MTP) observed among all records of occurrence of the species, which is represented by the value equal to 550. In other words, if a pixel, in any period or future scenario assessed, has a value of less than 550 in terms of climatic suitability, it is declared that the species would be locally extinct. This strategy is widely adopted to define critical thresholds because it has a strong biological support [43].

### Simulation of future spatial dynamics with MigClim

Unlike static models, which are used to evaluate the effects of step-by-step environmental changes, here we use the MigClim package [44] in R [33]. The main function of this package is to incorporate spatially explicitly the dispersion (movement) of the species over the study area as a function of events of changes in suitability for the time interval evaluated. In this sense, it was possible to quantify three fundamental results about the geographical distribution of a species under pressure of environmental changes (here climatic): [1] Persistence—when a particular pixel maintains, throughout the evaluated period, minimally satisfactory values of suitability that support the maintenance of the species; [2] Extinction—occurs when an evaluated pixel loses suitability for a critical climate threshold; & [3] Colonization—occurs when there is an increase in suitability, because of ongoing changes, in unoccupied pixels next to those that the species already occurred, causing the species to disperse to these neighboring pixels.

We used four environmental change events, (one for each period up to the end of the century) with two (2) dispersal events, equivalent to one event every 10 years. The scattering function for colonization of neighboring cells follows the following probabilistic values from the contiguous pixels: 1.0, 0.4, 0.16, 0.06, 0.03. This means that as the geographic departure of a particular pixel occurs, the likelihood of scattering events from that pixel decreases.

The main result of this spatial population dynamics simulation is a map where it is possible to map all the events described above (persistence, extinction, and colonization) and in which period such an event occurred. In addition, a special class of pixels is created where it can occur, until the end of the evaluation period, the resumption to a previous level of suitability. This class can be interpreted as those pixels that initially lost suitability for the critical level of extinction, but that eventually became favorable by the end of the simulation.

### Influence of altitude on the future spatial dynamics of *B*. *excelsa*

With the results of the spatialized simulations with MigClim for the different scenarios (extinction, colonization, and persistence), we tested the hypothesis that the colonization events for new areas could be correlated with altitude, given a visual pattern detected in the analyzes. In this sense, we extracted the altitude values for each cell of the map of the study area and tested the response of the simulations from a Pearson correlation analysis. Such analyzes were performed in R [33].

## Results

**Fig 1:**
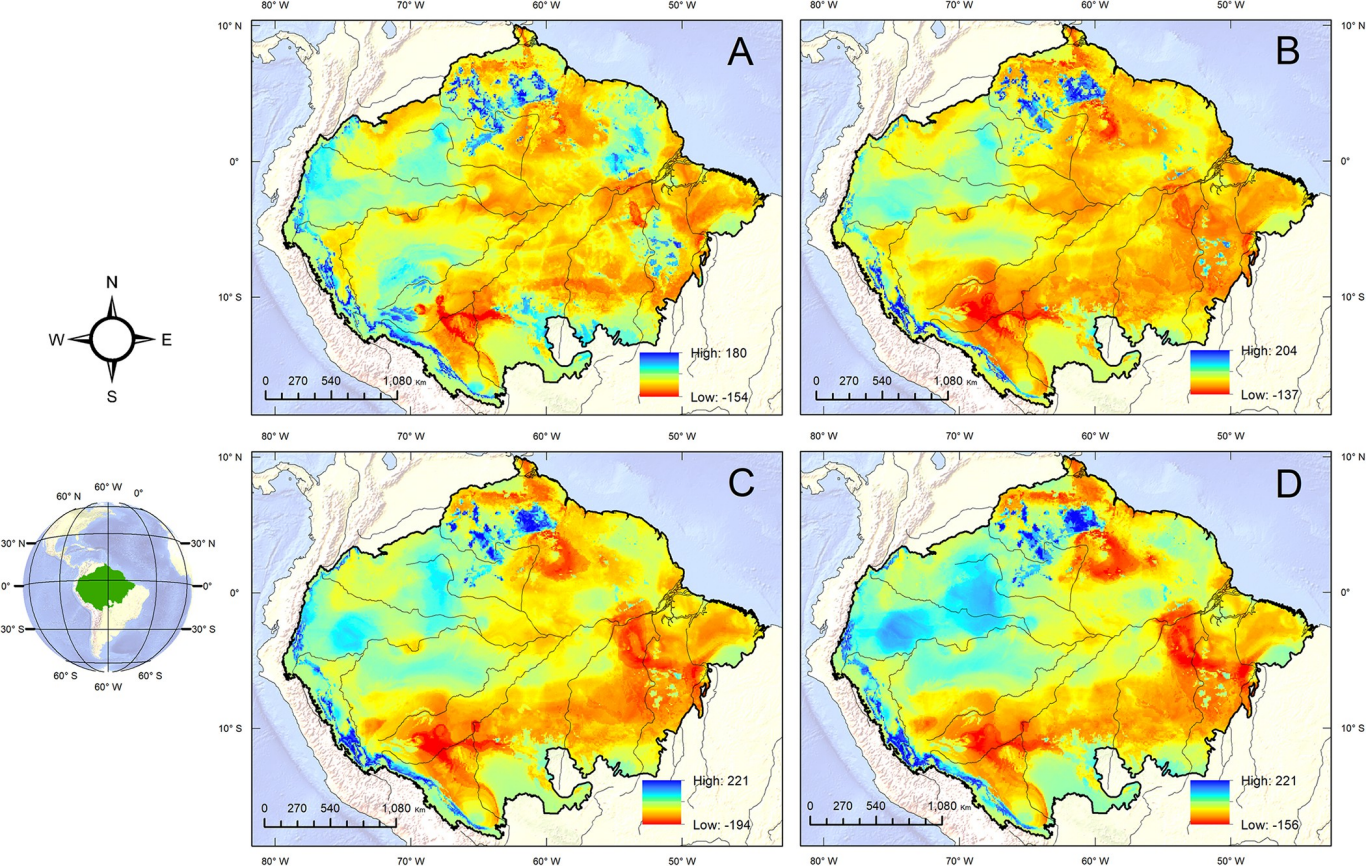
Gain or loss of climatic suitability for *Bertholletia excelsa* by the end of the century in the different climate scenarios evaluated. (A) SSP1-2.6; (B) SSP2-4.5; (C) SSP3-7.0; (D) SSP5-8.5. Made with Natural Earth. Free vector and raster map data @naturalearthdata.com.

The maps in Fig 1 show the trend of loss/gain of suitability from the present until the year 2100. The positive values (blue tones) show gain in suitability and the negative ones (yellow and red tones) show loss. We observed a loss of suitability for *Bertholletia excelsa* in most of the Amazon basin. This pattern is persistent among the different future scenarios evaluated. Some areas would be affected by a greater loss of climate suitability than others. For example, in the Southwest Amazon moist forests, in the region of the northern trough of the Amazon River—Uatuma-Trombetas moist forests—and a portion of the southeast—Tapajós-Xingu moist forests (areas in red). On the other hand, it was possible to observe that to the west and north of the Amazon basin, around the region of Japurá-Solimoes-Negro moist forests, there would be a gain in suitability, as well as those areas with higher altitude (Peruvian Yungas, Guianan moist forests, Xingu-Tocantins-Araguaia moist forests).

There are differences between future climatic scenarios that need to be highlighted. Although there is a tendency to reduce the generalized suitability for the Amazon basin, losing suitability would be more severe in the most pessimistic climate scenarios. This result is clear when the percentage lost via extinction in the different scenarios over time is evaluated (Table 1). All scenarios point to losses of more than 50% of the species’ distribution area due to climate change by the end of the century. In the SSP3-7.0 scenario, the reduction of the available area of the species could reach 94% of what is now the current distribution of *Bertholletia excelsa*. SSP2-4.5 would be the least severe among the future scenarios evaluated, with a loss of the original distribution area reaching 53.53% by the end of the century. Oppositely, the models predict that the gain in suitability tends to be more pronounced also toward the most pessimistic scenarios.

**Table 1:** Percentual of the potential distribution lost cumulatively via predicted extinction for each period (columns) and climate scenario (rows) analyzed in relation to the current distribution.

Period/Scenario	2021–2040	2041–2060	2061–2080	2081–2100
**SSP1-2.6**	41.11	55.68	59.92	62.29
**SSP2-4.5**	33.62	43.74	49.23	53.53
**SSP3-7.0**	44.51	73.27	88.39	94.37
**SSP5-8.5**	36.74	47.33	60.15	71.60

**Fig 2:**
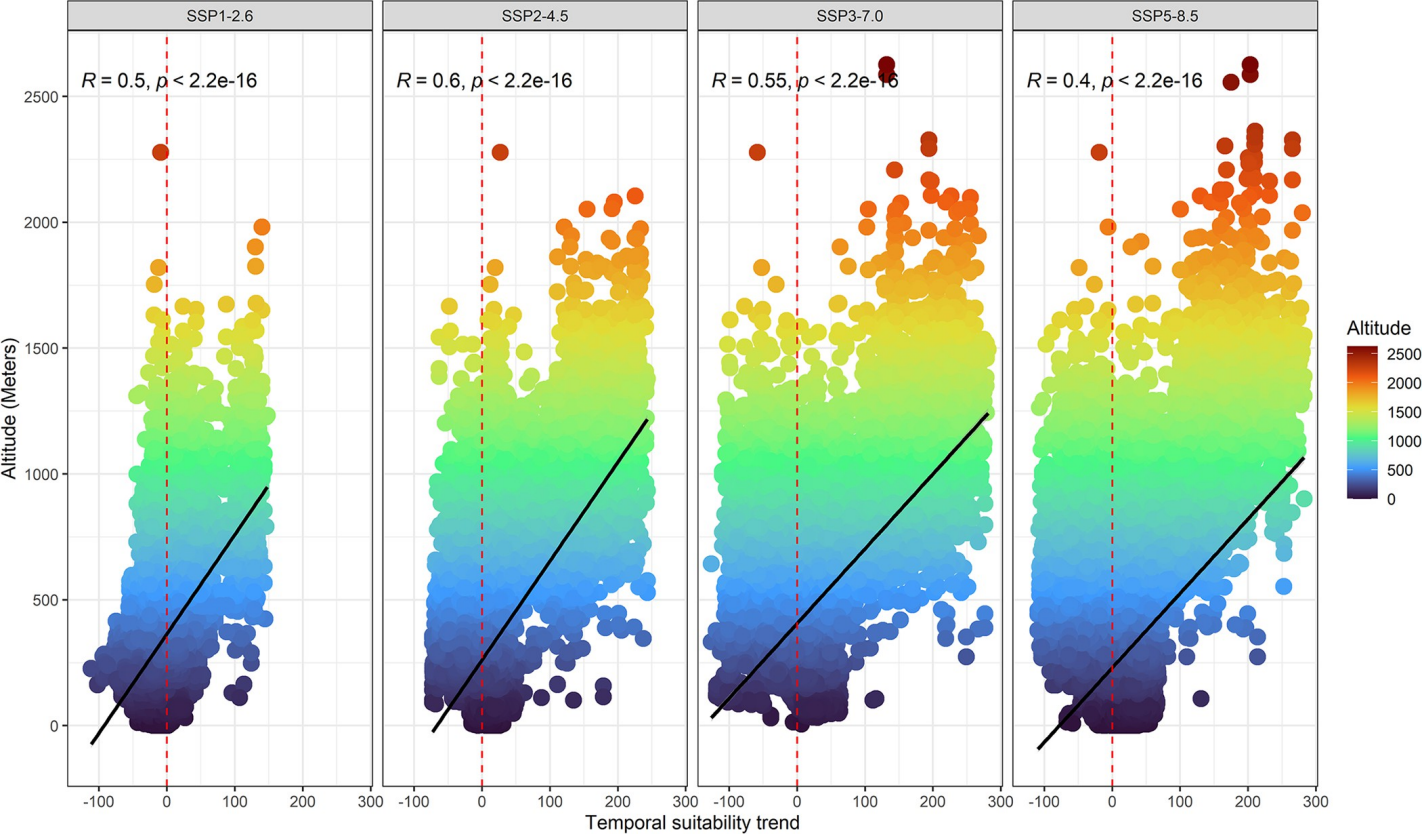
Pearson’s correlation between the trend of changes in suitability and altitude with the data of the simulated colonization events for new areas in the different scenarios. The dashed red line marks the division between the values of loss of suitability (left) and gain (right).

The graph in Fig 2 shows the relationship between the temporal trend of suitability and altitude. The results show that the colonization of the species to new areas may be related to a search for environments of higher elevation. This is evidenced by the positive values of the correlation between the tendency to increase suitability associated with the increase in altitude. It is also possible to observe that there are differences between the different climate scenarios. Apparently, the more severe the scenario (e.g., SSP5-8.5), the greater the effect of colonization to new areas of high elevation.

**Fig 3:**
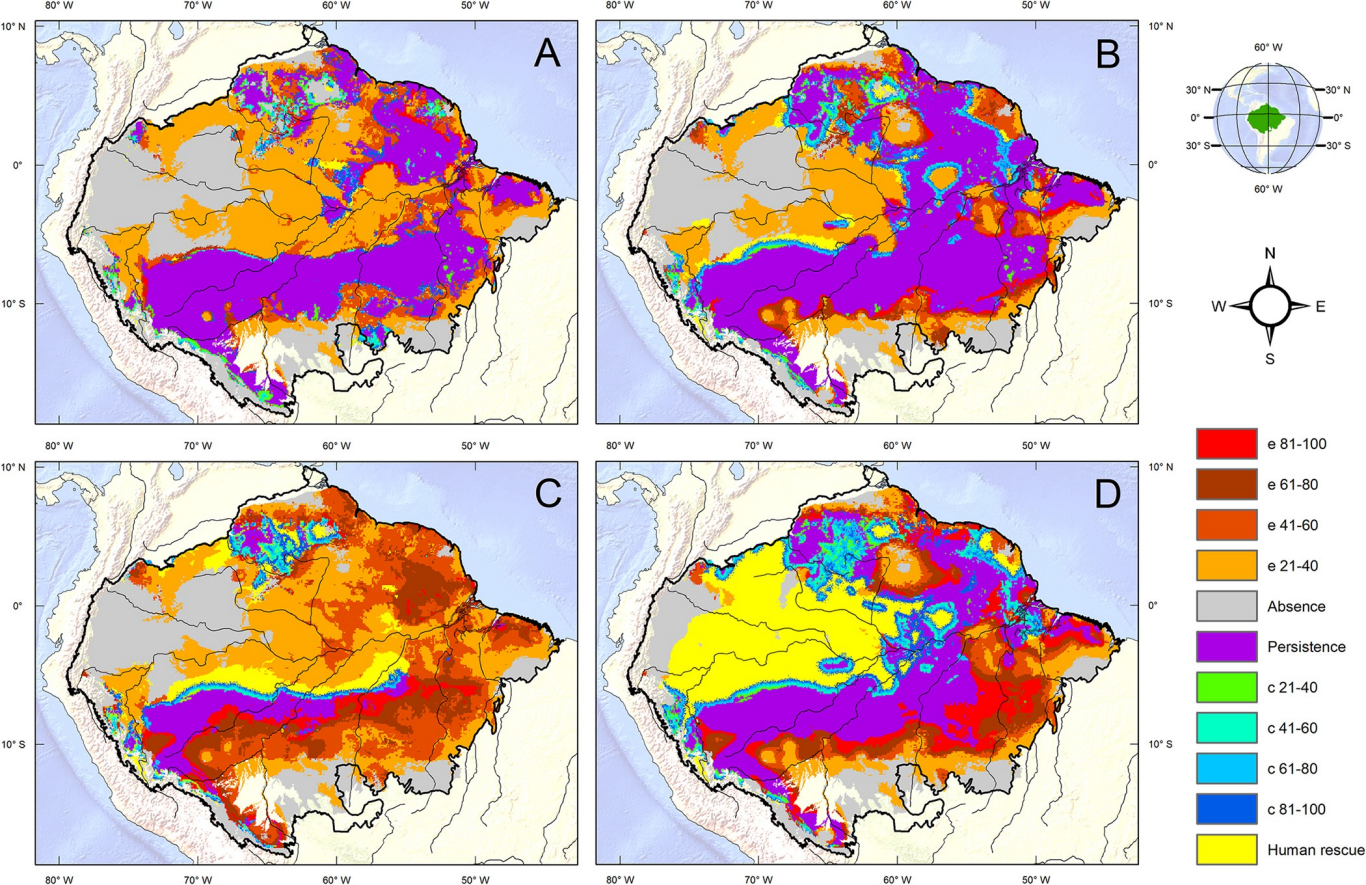
Spatial dynamics, considering extinction, colonization, and persistence, for *Bertholletia excelsa* in the different future climate scenarios evaluated in the study. Made with Natural Earth. Free vector and raster map data @naturalearthdata.com.

The maps in Fig 3 evaluate the spatial dynamics of the species in the different climatic scenarios. In the most optimistic scenario (Fig 3A), the local extinction of the species would occur in a large area in the first 20 years, being more severe than the scenario in Fig 3B (SSP2-4.5) B. The most dramatic situation would be in the SSP3-7.0 scenario (Fig 3C), in which the area where the species could persist is restricted to a narrow strip in the central Amazon towards the southwestern Amazon. In the most pessimistic scenario (Fig 3D), there would be a significant loss of habitat with virtual local extinction, mainly in the last period evaluated (areas in red).

Another relevant result observed concerns the possibility of reversing habitats that would become climatically unsuitable in the near future. Our results suggest that some areas in Fig 3 (in yellow) would become climatically suitable in the last 20 years of the century, specifically between 2080 and 2100. However, *Bertholletia excelsa* could not access them due to the lack of landscape connectivity, defined based on the parameters of the migration model for the species. They would be areas suitable climatically, but inaccessible to the species via natural dispersal. These would be areas that humans could manage the species, considering recovery from active ecological restoration. This pattern is most pronounced in the most pessimistic scenarios (SSP3-7.0 and SSP5-8.5).

## Discussion

The biogeographic distributions of trees in the Amazon are shaped by a combination of factors, such as drought tolerance, biological interactions, edaphic conditions, shade tolerance, and dispersal. Moreover, drought acts more strongly as an environmental filter for adult trees [45, 46]. Plants subjected to higher atmospheric concentrations of CO_2_ and increased air temperature show changes in photosynthetic production, resulting in changes in their ecophysiology [47, 48]. Thus, the risk of extinction of the Brazil nut may increase due to its sensitivity to environmental variations [17, 49].

Esquivel-Muelbert et al. investigated whether the floristic and functional composition of intact lowland Amazonian forests has been changing by evaluating records from 106 long‐term inventory plots spanning 30 years [50]. One of their results indicated that there had been notable increases in the relative abundance of the dry‐affiliated genera *Bertholletia*. One of the aggravating factors also for the effective success of the chestnut tree is its reproduction dependent on bee-mediated fertilization and the loss of these pollinators affects the long-term viability of chestnut populations [20]. In addition, Brazil nuts are also dependent on agouti for seed dispersal [51].

Inasmuch, climate change alters the geographic distribution of species and the response of each species depends on its individual intrinsic tolerance [52]. This displacement may lead to the loss of key relationships between species, contributing to the extinction of these biotic interactions [53]. Seed dispersal and pollination are closely dependent on animal-plant interactions for reproduction [53]. According to Sales et al. [54] found that the resilience of the biotic interactions between the Brazil nut tree and its community of dispersers may suffer a decoupling due to the niche mismatch for the year 2090. This mismatch was more frequent in the scenario more realistic scenario of climate change (SSP3-7.0).

In our results, we verified that the colonization regions are concentrated in areas of higher altitudes. These results are comparable with other works. For example, [55] found that 20% of the epiphyte species found in the Atlantic Forest will have their climatic niche shifted to higher altitudes. The same was observed by [56], who, working with ferns and lycophytes in Honduras, found that 28–53% of these will have altitudinal displacement by the year 2100 for both RCP 2.6 and RCP 8.5, respectively. [57] found that 47% of the fern and lycophyte species found along the mountain of the Celaque National Park, the highest in Honduras, will have to shift their distribution above the maximum altitude of the mountain under the scenario from RCP2.6 to the year 2050.

This displacement to areas of higher altitudes is also being recorded for animal species. Stream fish species of the genera *Barbus* and *Leuciscus*, it is predicted to colonize newly suitable areas in stretches of higher elevation or to extend their ranges towards the poles [58–60]. [61] studying birds endemic to the mountains of eastern Brazil predicted that all species studied in all scenarios (2050 and 2070) will experience a shift in their distributions to higher areas. A similar finding was also found for birds from high mountains in temperate and tropical regions, such as the Andes [62–64].

In many parts of its distribution range, *B*. *excelsa* occurs in an aggregated pattern, forming clusters known as ’manchales’, ’castanhais’ or ’reboleiras’, which are interspersed with vast areas of forest with very few or no trees. The scale of these clusters varies, and at the local level, the dispersion of *B*. *excelsa* is usually attributed to *Dasyprocta* species. At a regional and continental level, it is attributed to humans acting as dispersal agents, with evidence of human activity ranging from 12,000 to 20,000 years Before Present (BP) [21].

In a more recent scenario, it is known that the *B*. *excelsa* distribution across Central Amazonia changed drastically not only with regional climatic variability but also with major political and socioeconomic activities during post-colonial era (over 400 years ago)–the collapse of pre-Columbian indigenous (e.g., Mura populations) led to the interruption of management practices, affecting the *B*. *excelsa* as well [17].

In a future scenario where there is a reduction and/or absence of *Dasyprocta* species, *B*. *excelsa* will rely on human dispersion to colonize new areas suitable for its growth, as seen in the yellow areas in Fig 3. Moreover, future projections of climate change show that pollinators’ range may reduce drastically in the future, which might cause a spatial mismatch between *B*. *excelsa* and its community of pollinators [54]. In this sense, our finding aligns well with [21] results, especially for our SSP5-8.5 scenario result (Fig 3D), where the western portion of the Amazon might be suitable for *B*. *excelsa* colonization if there is human intervention.

It is difficult to predict how *B*. *excelsa* will fare in the future climate along with human intervention. However, because of its importance for the bioeconomy, it is possible to infer that the species might be planted in more areas towards the Brazilian Western Amazon in the future. Brazil is the leading exporter of Brazil nuts with shells, holding a 47% share of its global market between 2017 and 2019. The global market for this product is relatively small (US$24 million/year), and the main destinations for Brazil nuts with shells are Peru (38%) and Bolivia (16%). Both countries purchase Brazil nuts with shells as raw materials and use their superior industrial and commercial capacities to process and export them as Brazil nuts without shells, a product with a unit price four times higher (US$9.1/kg without shell against US$2.2/kg with shell) and a total market almost 15 times larger (US$364 million) [65].

Brazil nuts not only play a key role in the ecology and nutrient cycling of the Amazon forests, but also have their importance for human subsistence since the settlement of the Amazon [66, 67]. In addition, Brazil nuts play an important role in carbon sequestration [68]. In this way, information on the best areas for human management practices, restoration potential, contributes to more efficient conservation efforts, maintenance of Brazil nut trees and the design of management strategies, given that the species is currently considered threatened by the Ministry of the Environment (Law No. 443/2014 MMA, 2014).

## Conclusions

Our results indicate that there is a high risk of the extinction of the iconic species *Bertholletia excelsa* because of the effects of climate change by the end of the century. This risk can be reduced if the species can access areas of elevation via migration, as well as have an active recovery plan based on effective management of the species, especially in the most pessimistic climate scenarios.

## Supporting information

S1 Table Averaged (Mean) and Standard Deviation (SD) values for evaluation metrics (TSS and ROC) by different methods for specie distribution model. Average values were calculated utilizing 10 replicates.
(DOCX)

S2 Table Importance metrics of the predictors by different methods to build the specie distribution model. (DOCX)
