# Supplementary material for: Brazil nut journey under future climate change in Amazon
Source: PLoS One. 2024 Nov 13;19(11):e0312308. doi: 10.1371/journal.pone.0312308 (PMC11559973; doi:10.1371/journal.pone.0312308)
Supplement: S1 Table — Average values were calculated utilizing 10 replicates. (DOCX) [file pone.0312308.s001.docx]

**Supporting information**

**S1 Table.** Averaged (Mean) and Standard Deviation (SD) values for evaluation metrics (TSS and ROC) by different methods for specie distribution model. Average values were calculated utilizing 10 replicates.

| **Method** | **TSS - Testing data (Mean)** | **ROC - Testing data (Mean)** | **SD-TSS** | **SD-ROC** |
| --- | --- | --- | --- | --- |
| ANN | 0.61 | 0.84 | 0.03 | 0.03 |
| CTA | 0.61 | 0.81 | 0.03 | 0.02 |
| FDA | 0.61 | 0.85 | 0.05 | 0.03 |
| GAM | 0.64 | 0.86 | 0.04 | 0.03 |
| GBM | 0.66 | 0.89 | 0.04 | 0.02 |
| GLM | 0.61 | 0.82 | 0.04 | 0.03 |
| RF | 0.68 | 0.91 | 0.03 | 0.02 |
