# Supplementary material for: Brazil nut journey under future climate change in Amazon
Source: PLoS One. 2024 Nov 13;19(11):e0312308. doi: 10.1371/journal.pone.0312308 (PMC11559973; doi:10.1371/journal.pone.0312308)
Supplement: S2 Table — (DOCX) [file pone.0312308.s002.docx]

**Supporting information**

**S2 Table.** Importance metrics of the predictors by different methods to build the specie distribution model.

| **Method** | **bio1** | **bio7** | **bio15** | **bio12** |
| --- | --- | --- | --- | --- |
| ANN | 0.14 | 0.28 | 0.18 | 0.54 |
| CTA | 0.43 | 0.08 | 0.08 | 0.49 |
| FDA | 0.22 | 0.08 | 0.10 | 0.48 |
| GAM | 0.41 | 0.23 | 0.10 | 0.42 |
| GBM | 0.38 | 0.08 | 0.09 | 0.44 |
| GLM | 0.31 | 0.04 | 0.15 | 0.44 |
| RF | 0.39 | 0.20 | 0.17 | 0.41 |
| **Mean (All methods)** | 0.33 | 0.14 | 0.12 | 0.46 |
